# PRC1 Stabilizes Cardiac Contraction by Regulating Cardiac Sarcomere Assembly and Cardiac Conduction System Construction

**DOI:** 10.3390/ijms222111368

**Published:** 2021-10-21

**Authors:** Xixia Peng, Gang Feng, Yanyong Zhang, Yuhua Sun

**Affiliations:** 1 Institute of Hydrobiology, Chinese Academy of Sciences, Wuhan 430072, China; pengxixia1990@163.com (X.P.); fenggangscu@foxmail.com (G.F.); zhangyanyong0924@163.com (Y.Z.); 2 College of Advanced Agricultural Sciences, University of Chinese Academy of Sciences, Beijing 100049, China; 3 The Innovation of Seed Design, Chinese Academy of Sciences, Wuhan 430072, China

**Keywords:** Rnf2, PRC1, cardiac contraction, sarcomere assembly, cardiac conduction system

## Abstract

Cardiac development is a complex process that is strictly controlled by various factors, including PcG protein complexes. Several studies have reported the critical role of PRC2 in cardiogenesis. However, little is known about the regulation mechanism of PRC1 in embryonic heart development. To gain more insight into the mechanistic role of PRC1 in cardiogenesis, we generated a PRC1 loss-of-function zebrafish line by using the CRISPR/Cas9 system targeting *rnf2*, a gene encoding the core subunit shared by all PRC1 subfamilies. Our results revealed that Rnf2 is not involved in cardiomyocyte differentiation and heart tube formation, but that it is crucial to maintaining regular cardiac contraction. Further analysis suggested that Rnf2 loss-of-function disrupted cardiac sarcomere assembly through the ectopic activation of non-cardiac sarcomere genes in the developing heart. Meanwhile, Rnf2 deficiency disrupts the construction of the atrioventricular canal and the sinoatrial node by modulating the expression of *bmp4* and other atrioventricular canal marker genes, leading to an impaired cardiac conduction system. The disorganized cardiac sarcomere and defective cardiac conduction system together contribute to defective cardiac contraction. Our results emphasize the critical role of PRC1 in the cardiac development.

## 1. Introduction

As the first organ to generate and function in the embryo, heart development is strictly regulated by various molecules and signal pathways. To become mature and functional, the embryonic heart undergoes a series of complex processes, including the specification and differentiation of cardiac lineages, the morphogenesis of the heart tube, looping, the assembly of cardiac chambers, the constriction of the atrioventricular canal, and the establishment of proper cardiac functions [1,2,3]. Errors in any of these processes can cause congenital heart malformations [4,5].

The main function of the heart is to drive blood circulation with sturdy contractions triggered by electrical impulses. Defects in cardiac function are often due to abnormalities in the cardiac contractile apparatus or the cardiac conduction system (CCS) [3]. The cardiac contractile apparatus, namely the cardiac sarcomere, provides a structural basis for cardiac contraction [6]. The cardiac sarcomere is mainly composed of two filamentary systems, the thick and thin filaments. The myosin-based thick filaments constitute the electron-dense A-bands, whereas the actin-based thin filaments make up the least electron-dense I-bands. The M-lines, anchoring the bipolar thick filaments, are located in the middle of the A-bands. The Z-disc, located in the middle of the I-bands and anchoring the end of the thin filaments and the third filament system in the sarcomere-titin filaments, defines the boundary between two adjacent sarcomere units [6,7,8]. The development of a functional cardiac sarcomere requires promoting the expression of cardiac sarcomere genes and repressing the expression of non-cardiac sarcomere genes in the heart. The ectopic activation of non-cardiac myofibril genes in the developing heart can disrupt cardiac sarcomere formation and cause impaired cardiac function [9].

The CCS is responsible for initiating and propagating proper electrical impulses to generate and maintain coordinated cardiac contraction [10]. The sinoatrial node (SAN) and the atrioventricular canal (AVC) are two critical components of the CCS [10]. The specialized pacemaker myocyte in SAN initiates the electrical impulse. The AVC, where the atrioventricular node and the atrioventricular bundle are located, is critical for the conduction delay and electrical impulse propagation that lead to the coordinated contraction of the atrium and ventricle [10]. The specification of the AVC can be defined by the restricted expression of *notch receptor 1b* (*notch1b*), *hyaluronan synthase 2* (*has2*), and *activated leukocyte cell adhesion molecule a* (*alcama*) in the endocardium, as well as *T-box transcription factor 2b* (*tbx2b*), *bone morphogenetic protein 4* (*bmp4*), and *versican a* (*vcana*) in the myocardium [3,11]. Multiple AVC marker genes present a specific change in the expression region from throughout the anteroposterior extent of the heart to specific cells within the AVC, such as *bmp4*, *vcana*, and *notch1b* [11,12]. Expanded expression of AVC marker genes can cause AVC malformation and result in a dysmorphic heart with disordered cardiac contraction [13,14]. The gene *bmp4* is also expressed in pacemaking cells in the SAN, targeted directly by the homeodomain transcription factor Shox2, which is essential for sinoatrial valve formation and pacemaking system development [15,16].

As well-known epigenetic regulators [17], the Polycomb Group (PcG) proteins are involved in a plethora of biological processes, including epigenetic inheritance [18], X-chromosome inactivation [19,20], stem cell self-renewal [21], senescence [22], and tumorigenesis [23,24,25]. The PcG proteins primarily form two principal complexes, Polycomb-repressive complex 1 (PRC1) and Polycomb-repressive complex 2 (PRC2). PRC2 is responsible for the trimethylation of Lys27 on histone H3 (H3K27me3) via the EZH2 or EZH1 protein subunit, while PRC1 catalyzes the ubiquitination of Lys119 on histone H2A (H2AK119ub) through E3 ligase RING1 [26,27]. Although PRC1 complexes show high complexity in their composition, RING1 is shared by all PRC1 subfamilies [28]. Two *RING1* homologues (*Ring1a* and *Ring1b*/*Rnf2*) located in the mice genomes, and both of them can assemble into PRC1 [28]. Ablating RING1A and RNF2 simultaneously lead to lethal aplasia in mice [26]. Only one *RING1* ortholog, namely *rnf2*, exists in the zebrafish genomes. The *rnf2* mRNA distribution was not spatially restricted during development, with obvious expression in the brain and pectoral fin [29]. The deletion of Rnf2 induces severe developmental defects in zebrafish embryos, including craniofacial abnormality and the absence of pectoral fins [29,30]. A recent study has shown that Rnf2 may regulate cardiac looping by repressing *tbx2/3* expression [31]. However, the conclusion was mainly drawn from 3 dpf embryos. The mechanism through which Rnf2 regulates early cardiogenesis remains elusive. 

To obtain further insight into the role of PRC1 in early cardiogenesis, we generated zebrafish Rnf2 mutants by using the CRISPR/Cas9 system. We found that Rnf2 is crucial to maintaining regular cardiac contraction. Rnf2 loss-of-function disrupts cardiac sarcomere assembly and CCS construction by dysregulating specific transcription programs. Our results emphasized the critical role of PRC1 in the development of zebrafish embryonic hearts.

## 2. Results

### 2.1. Loss of Rnf2 Caused Embryonic Lethality with Severe Cardiac Defects

**Figure 1:**
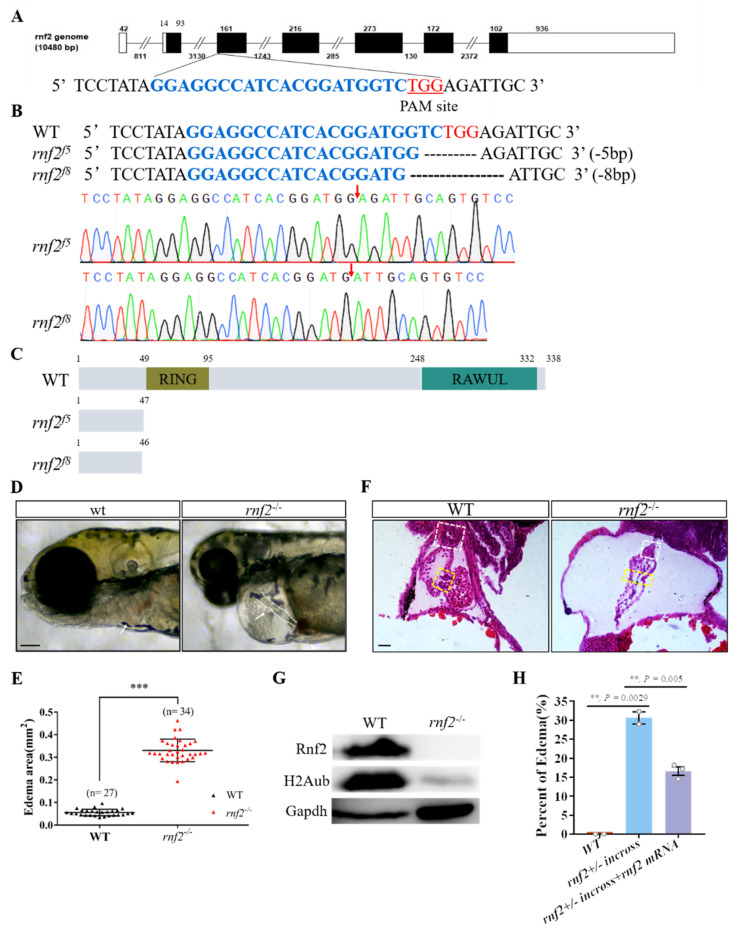
Rnf2-null zebrafish mutant displayed severe cardiac defects. (**A**) The target site (sequence highlighted in blue) is located in exon 3. The PAM site is underlined and highlighted in red; (**B**) DNA sequencing identified that two mutant alleles carried a 5 bp (*rnf2*^f5^) and 8 bp (*rnf2*^f8^) deletion respectively. The deletion sites are indicated by red arrows; (**C**) Schematic diagram of the wild-type and mutant Rnf2 proteins. Zebrafish wild-type Rnf2 contained an N-terminal Ring-finger domain (yellow) and a C-terminal RAWUL domain. The two mutant proteins were truncated before the RING-finger domain; (**D**) The *rnf2^−/−^* larvae displayed severe pericardia edema. The hearts are indicated by white arrows, the stringy heart in *rnf2^−/−^* is depicted by white dotted lines, scale bar: 0.2 mm; (**E**) Scatter plot showing the sectional area of edema in wild-type (black) and *rnf2^−/−^* (red). (**F**) Histological sections of 4 dpf heart, boxes in yellow indicate the AVC and valves, white boxes indicate the bulbous arteriosus, scale bar: 50 μm, n(WT) = 2, n (*rnf2^−/−^*) = 3; (**G**) Western blot verified the deletion of Rnf2 protein. Gapdh was set as internal reference; (**H**) Bar graph showing the percentage of embryos with edema. **, *p* < 0.01; ***, *p* < 0.001; n, sample number.

To investigate the role of PRC1 in embryonic development, we generated an Rnf2 mutant zebrafish line using the CRISPR/Cas9 system. The exon 3 of *rnf2* was targeted and two mutant alleles (*rnf2*^f5^ and *rnf2*^f8^) were generated, as revealed by genotyping (Figure 1A,B). The two mutant alleles were predicted to encode the truncated proteins lacking both the RING and RAWUL domains (Figure 1C). No obvious difference was found between the control and mutant embryos at 24 hpf, suggesting that Rnf2 is not required for the formation of a basic body structure in zebrafish. However, *rnf2^−/−^* embryos can be separated from their *rnf2^+/+^* and *rnf2^+/−^* siblings morphologically as early as 36 hpf, as the mobility of *rnf2^−/−^* embryos is reduced [29]. At 72 hpf, *rnf2^−/−^* embryos exhibited pleiotropic phenotypes, including defective craniofacial structures, small eyes, missing pectoral fins and clear pericardial edema encompassing a stringy heart with weak contractility (Figure 1D,E and Figure S1), which was consistent with previous reports [29,30,31]. The *rnf2^−/−^* larvae usually die within a week, most likely of severe heart failure.

To gain a more detailed view of the cardiac defects in *rnf2^−/−^* embryos, we performed histological analyses on the hearts of 4 dpf embryos. The results showed that the heart in the *rnf2^−/−^* embryos was spindly, with fusiform atriums and ventricles. Unlike the heart of the wild-type embryos, which displayed a constrictive AVC between the atrial and ventricular chambers and protruded well-formed primitive valve leaflets, the AVCs of the *rnf2^−/−^* embryos were less constricted and the primitive valve leaflets were missing (Figure 1F). These results suggested that the formation of the AVC and primitive valves are severely disrupted in *rnf2^−/−^* embryos.

To verify that the cardiac defects resulted from the functional deficiency of Rnf2, we first collected the assumptive *rnf2^−/−^* embryos with pericardial edema and stringy hearts and investigated their Rnf2 protein levels through western blotting. As we expected, unlike the wild-type embryos showing high Rnf2 levels, no Rnf2 was detected in the assumptive *rnf2^−/−^* embryos, and H2AK119ub1 enrichment was also decreased to an almost undetectable level (Figure 1G). The residual H2AK119ubi might have been contributed by maternal Rnf2. We also performed rescue assays by microinjecting exogenous *rnf2* mRNA into the 1-cell-stage embryos from *rnf2^+/−^* self-cross and counted the embryos with and without pericardial edema at 72 hpf. The results showed that the overexpression of *rnf2* reduced the percentage of embryos with edema from 32% to 17% (Figure 1H). As expected, the percentage of embryos with the stringy heart phenotype was also reduced to a similar extent. These data strongly suggested that the observed heart defects were caused specifically by Rnf2 loss-of-function.

### 2.2. The Cardiac Contraction Was Disrupted in the Rnf2-Null Embryos

**Figure 2:**
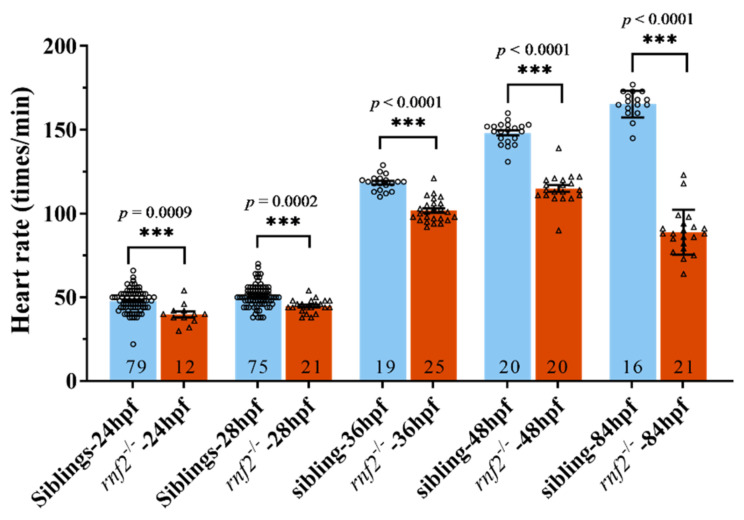
The cardiac contraction was disrupted in Rnf2-null zebrafish embryos. Scatter plot showing the heart rates of wild-type (blue) and *rnf2^−/−^* (red) embryos at different developmental stages. Numbers in the bottoms of the bars indicate the sample number of each group. ***, *p* < 0.001.

It was observed that the hearts of the *rnf2^−/−^* embryos contracted much slower than those of the wild-type at 72 hpf. To determine when the cardiac contractions began to be affected, we measured the heart rates of the *rnf2^−/−^* and wild-type embryos at different developmental stages. In the wild-type embryos, the heart started to contract by about 20 hpf and sped up with development (Figure 2). The hearts of the *rnf2^−/−^* embryos started to contract by about 20 hpf as well, but the heart rates increased more slowly, leading to a reduced heart rate at 24 hpf (Figure 2). Furthermore, the heart rates of the *rnf2^−/−^* embryos even decreased at 84 hpf (Figure 2). These data showed that the cardiac contraction of *rnf2^−/−^* embryos was affected as early as 24 hpf, indicating that defects in cardiac development began no later than 24 hpf.

### 2.3. The Mesoderm Formed Normally in the Rnf2-Null Zebrafish Embryos

**Figure 3:**
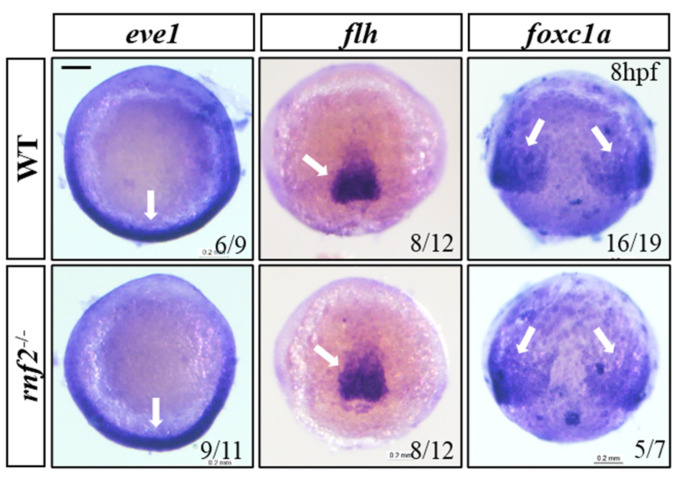
The mesoderm formed normally in Rnf2-null zebrafish embryos. WISH results showing expression of mesoderm markers *eve1* (ventral mesoderm), *flh* (axial mesoderm), and *foxc1a* (paraxial mesoderm). The expression regions are indicated by white arrows. Scale bar: 0.2 mm.

During the cardiogenesis process, both cardiomyocytes and endocardial cells originate in the mesoderm [32,33,34]. Since the cardiac contractions of the *rnf2^−/−^* embryos were affected just as the hearts started beating, we wondered whether the cardiac development became defective even earlier. To investigate whether the cardiac defects resulted from aberrant mesoderm formation, we examined the expression of the representative mesoderm markers *eve1* (ventral mesoderm), *flh* (axial mesoderm), and *foxc1a* (paraxial mesoderm) at 8 hpf by using WISH. The WISH results showed that these mesoderm markers expressed normally in the absence of Rnf2 (Figure 3), indicating that mesoderm formation was not affected in the *rnf2^−/−^* embryos.

### 2.4. The Heart Tube Structure Appeared Normal in the rnf2^−/−^ Embryos

**Figure 4:**
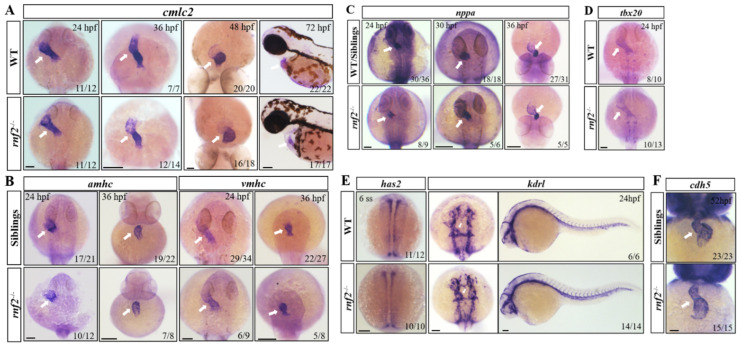
Expression of cardiomyocyte markers examined by in situ hybridization. (**A**) *cmlc2* at different developmental stages; (**B**) Atrial (*amhc*) and ventricle (*vmhc*) cardiomyocyte markers at 24 hpf and 36 hpf; (**C**) Growth factor *nppa* at 24 hpf, 30 hpf, and 36 hpf; (**D**) *tbx20* at 24 hpf; (**E**) Expression of endocardial precursor marker gene *has2* and endothelial marker gene *kdrl* in the heart and blood vessels, white arrows indicate the heart tube; **(F**) Expression of endothelial marker gene *cdh5* in the heart. The expression of genes in the heart is indicated by white arrows. Scale bar: 0.2 mm.

Given the normal mesoderm formation, we wondered whether the cardiac progenitor specification, migration, and heart tube generation were affected in the *rnf2^−/−^* embryos. We first examined the expression of pan-myocardial gene *cardiac myosin light chain 2* (*cmlc2*) at different developmental stages. The results showed that at 24 hpf and 36 hpf, the expression of *cmlc2* in the *rnf2^−/−^* embryos displayed no obvious difference from that of the wild-types (Figure 4A). At 48 hpf and 72 hpf, except for the alteration in the shape of the expression region, which may have been due to variations of the heart shape, the transcription level of *cmlc2* still showed no obvious difference (Figure 4A). We then detected the expression of the atrial and ventricular myocardium-specific genes *atrial myosin heavy chain* (*amhc*) and *ventricular myosin heavy chain* (*vmhc*). The expression of *amhc* and *vmhc* showed no significant anomalies (Figure 4B). The expression of the cardiac regulation genes *tbx20* and *natriuretic peptide A* (*nppa*) was normal as well (Figure 4C,D). These results indicated that the myocardial specification and migration were largely normal in the *rnf2^−/−^* embryos.

The heart tube is composed of an outer myocardial layer and an inner endothelial layer at early stages. We also examined the expression of the endothelial marker *has2* at 6-somite stage, as well as *kinase insert domain receptor-like* (*kdrl*) at 24 hpf and *cadherin 5* (*cdh5*) at 52 hpf. The results showed the normal expression of all these three genes, indicating that the endocardium largely formed normally in the *rnf2^−/−^* embryos (Figure 4E,F). These results suggested that cardiac progenitor specification and migration are not strictly dependent on Rnf2, and that the heart tube formation appeared normal in the *rnf2^−/−^* embryos.

### 2.5. Rnf2 Deficiency Disorganized the Sarcomere Assembly in Zebrafish Hearts

**Figure 5:**
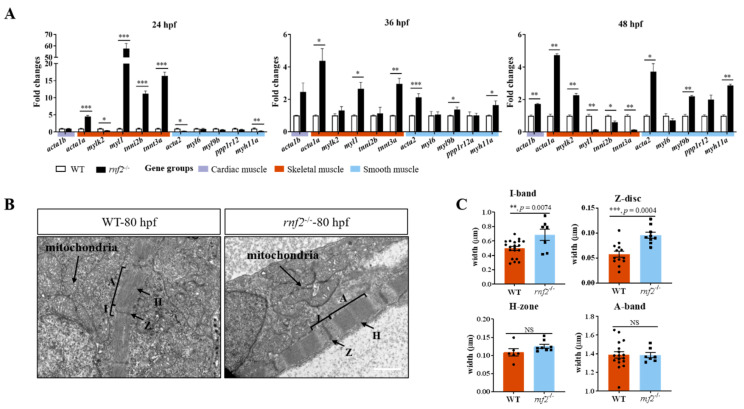
Rnf2 deficiency disorganized sarcomere assembly in zebrafish hearts. (**A**) Real-time PCR tested the expression of skeletal and smooth muscle genes at 36 hpf (left) and 48 hpf (right). The fold changes of relative mRNA levels are presented as mean ± SEM. The expression in wild-types was normalized to 1. The experiment was repeated on three separate occasions. n = 159, 111, and 161 for *rnf2^−/−^* group at 24 hpf, 36 hpf, and 48 hpf, respectively; n = 145, 113, and 164 for WT group at 24 hpf, 36 hpf, and 48 hpf, respectively. (**B**) Cardiac TEM revealed the sarcomere of cardiac muscle was abnormal in *rnf2^−/−^* hearts. A, A-band; I, I-band; H, H-zone; Z, Z-disc. Scale bar: 1.0 μm. n = 3. (**C**) Bar graph showing the width of A-band, I-band, Z-disc, and H-zone in wild-type and *rnf2^−/−^* cardiac sarcomeres. *, *p* < 0.05; **, *p* < 0.01; ***, *p* < 0.001; NS, no significant.

Cardiac contraction deficiency is often due to abnormalities in the cardiac contractile apparatus or the CCS [3]. The cardiac sarcomere is the contractile unit of the heart, providing a structural basis for cardiac contraction [6]. To verify whether the cardiac sarcomere assembly was disrupted in the *rnf2^−/−^* embryos, we first detected the transcription of genes associated with non-cardiac myofiber, including skeletal and smooth muscle myofibers, in the embryonic hearts. Compared with the controls (wild-type or sibling embryos), the expression of skeletal and smooth muscle genes was dysregulated in the hearts of the *rnf2^−/−^* embryos (Figure 5A). At 24 hpf, the expression of the skeletal muscle genes *acta1a*, *myl1*, *tnni2b*, and *tnnt3a* was significantly upregulated, while the expression of the cardiac sarcomere gene *acta1b* and the smooth muscle genes *myl6*, *myl9b*, and *ppp1r12* was not affected (Figure 5A). Later on, the transcription levels of cardiac *acta1b* and the smooth muscle genes *acta2*, *myl9b*, *ppp1r12*, and *myh11a* were elevated gradually and all were upregulated significantly at 48 hpf, while the expression of skeletal muscle genes was reduced gradually and downregulated significantly at 48 hpf (Figure 5A). These results prompted us to deduce that the cardiac sarcomere of the *rnf2^−/−^* embryos may have been defective. To verify this deduction, we further examined the cardiac structure of the wild-type and mutant embryos by using TEM. The results showed that the cardiac sarcomere structure of the *rnf2^−/−^* embryos was different from that of the wild-types (Figure 5B). The myofibril fibers in the *rnf2^−/−^* embryos were arranged more tightly. The I-band and Z-disc in the *rnf2^−/−^* hearts were significantly wider than in those of the wild-type embryos (Figure 5C). Thus, we propose that the loss of Rnf2 may trigger defective cardiac sarcomere alignment via the ectopic transcription activation of non-cardiac sarcomere genes in the developing heart, contributing to severe cardiac contractile defects in *rnf2^−/−^* embryos.

### 2.6. Rnf2 Deficiency Caused Defects in the Cardiac Conduction System

**Figure 6:**
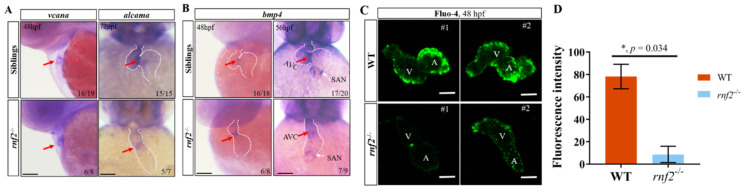
Rnf2 deficiency caused defects in the cardiac conduction system. (**A**) Expression of AVC myocardial markers *vcana* and *alcama*; (**B**) Expression of AVC endocardial and SAN marker *bmp4*. Red arrows indicate AVC; (**C**) Fluo-4 staining detects calcium signal intensity in the hearts of *rnf2^−/−^* or wild-type embryos; V, ventricle; A, Atrium; #1/#2, sample number; n = 2. (**D**) Quantification of fluorescence intensity in Figure 6C. *, *p* < 0.05; Scale bar: 0.2 mm.

As well as abnormalities in the contractile apparatus, the deficiency in the CCS can cause contraction defects. Histological analysis showed that the AVC in the *rnf2^−/−^* embryos was not as constrictive as in the wild-type embryos. The AVC is a core component of the CCS; this prompted us to speculate that the CCS in the *rnf2^−/−^* embryos may be defective. To confirm this speculation, we first examined the AVC patterning by detecting the expression of *vcana* and *alcama* in *rnf2^−/−^* embryos by using WISH (Figure 6A). In the control embryos, the AVC was well formed between the atrial and ventricular chambers, and the expression of *alcama* and *vcana* was restricted to the AVC region. By contrast, the *rnf2^−/−^* embryos lacked a clear AVC ring, and the mRNA distribution of *alcama* and *vcana* was diffused and extended into the flanking chambers, instead of being restricted to the AVC (Figure 6A). These results indicated that the AVC constriction was disrupted in the hearts of the *rnf2^−/−^* embryos. Furthermore, we detected the expression of *bmp4* at 48 hpf and 56 hpf in the *rnf2^−/−^* embryos (Figure 6B). With a shorter staining time of 48 hpf embryos, the expression of *bmp4* at the AVC was expanded in the *rnf2^−/−^* embryos, as was that of *alcama* and *vcana* (Figure 6B). Furthermore, at 56 hpf, with a longer staining time, the expression of *bmp4* at SAN was clearly reduced (Figure 6B), indicating that the specialized pacemaker myocytes in SAN were decreased. These data suggested that the CCS was disrupted in the *rnf2^−/−^* embryos.

Calcium signaling is a crucial indicator of CCS function. To map the cardiac conduction optically, we monitored the calcium signal intensity in individual embryonic hearts using a calcium-sensitive dye, fluo-4 AM [35]. Our results showed that the calcium signal intensity in the hearts of the *rnf2^−/−^* embryos was dramatically reduced when compared with that of the wild-types (Figure 6C,D), which further supported the argument that Rnf2 deficiency disrupted the CCS in the embryonic hearts, indicating the crucial role of Rnf2 in zebrafish CCS construction.

Taken together, our results suggested that Rnf2 helps to generate proper cardiac contraction by regulating the expression of genes associated with cardiac sarcomere assembly and the cardiac conduction system.

## 3. Discussion

Cardiac development is a complex process strictly controlled by various factors, including the PcG protein complexes. Cardiac-conditional EZH2 deficiency causes severe cardiac defects, including persistent hypertrabeculation, right ventricular hypoplasia, atrial and ventricular septal defects, myocardial fibrosis, and moderately impaired left ventricular systolic function [36]. Zebrafish ezh2 mutant embryos also displayed cardiac differentiation defects, leading to stringy hearts [37]. Reducing embryonic ectoderm development protein (EED), a crucial component of PRC2, in developing cardiomyocytes leads to lethal cardiogenesis defects [36]. These works indicated the critical role of epigenetic repression established by PRC2 in cardiogenesis. Similarly, PRC1 subunits, such as Ring1b, Bmi1/Pcgf4, and Rae28/Phc1, were also reported as playing crucial roles in cardiac development [38,39,40,41]. The simultaneous deletion of RNF2 and RING1A results in the loss of function of the entire PRC1 family. However, the functional ablation of mouse Rnf2 causes gastrulation arrest and results in embryonic lethality [42], which prevented us from investigating the function of PRC1 in cardiogenesis. Rnf2^I53A/I53A^ mouse embryos, which ablate the E3 ligase activity but retain the ability to incorporate RNF2 complexes into PRC1, exhibit pericardial edema at E15.5, suggesting defects in the developing cardiovascular system [43,44]. Zebrafish Rnf2 was reported to regulate cardiac looping by repressing the transcription of *tbx* genes [31]. Through single-heart RNA-seq analysis, Chrispijn et al. showed that *tbx2/3* were upregulated in the hearts of Rnf2 mutants at 2 dpf and/or 3 dpf (but not 1 dpf) [31]. However, we found that the heart rates of *rnf2^−/−^* embryos were reduced significantly as early as 24 hpf, indicating that cardiac contraction was disrupted at no later than 24 hpf. Our data suggest that Rnf2 may be involved in different processes of zebrafish cardiogenesis through the epigenetic regulation of different transcriptional programs. This inference is consistent with the consensus repression mechanisms by which PcG complexes control the transcription of target genes through chromatin compaction and genome-wide histone modification [45,46,47], and is substantiated by our finding that Rnf2 regulates zebrafish cardiac contraction by stabilizing proper transcription programs of sarcomere genes and CCS associated genes in the heart.

1. PRC1 maintains regular cardiac sarcomere assembly by orchestrating the sarcomere gene transcription program.

The patterning of the embryonic heart requires proper cardiomyocyte differentiation and the assembly of specific cell types into a functional apparatus. The cardiac sarcomere is the basic contractile unit of myofibrils in the heart [48], forming the structural foundation of cardiac contraction. Disturbance of the cardiac sarcomere structure disrupts contractile activity and causes heart failure phenotypes [36,49,50]. The correct generation of a functional cardiac sarcomere requires the activation of cardiac myofibril structural genes and, simultaneously, repression of the expression of non-cardiac sarcomere paralogs in the developing heart. Such extensive orchestration of transcription coincides with the mechanisms of epigenetic transcription control. Indeed, nucleosome remodeling and the deacetylase (NuRD) and PRC2 complexes were both reported as being involved in this transcriptional control, directly or indirectly [9,36,50]. CHD4, the catalytic core of the NuRD complex, regulates skeletal- and smooth-muscle-specific transcription programs by directly binding to their promoters. The cardiac conditional null of CHD4 disrupted the cardiac sarcomere structure by inducing the ectopic expression of non-cardiac sarcomere genes in the developing heart [9]. EZH2, the core catalytic subunit of PRC2, represses the transcription of non-cardiac muscle genes in the developing heart by repressing the expression of Six1, which serves to activate the transcription of skeletal muscle genes [36,50]. However, the role of PRC1, another crucial repressive epigenetic regulator, in this transcription control has not been reported. We propose here that the PRC1 complex also plays a critical role in stabilizing the transcription program of sarcomere genes in the developing heart. A group of non-cardiac muscle genes were misexpressed in the developing hearts of Rnf2 mutants. At 24 hpf, the skeletal sarcomere structural genes *acta1a*, *myl1*, *tnni2b*, and *tnnt3a* were all upregulated. The ectopic activation of skeletal muscle genes in the early heart disrupts the cardiac sarcomere, as revealed by TEM, leading to cardiac contraction deficiency. We conclude that PRC1 regulates cardiac sarcomere assembly by repressing the expression of non-cardiac sarcomere genes in the developing heart.

2. PRC1 represses sarcomere genes indirectly.

In another manuscript, which is currently under preparation, we performed a ChIP-seq analysis of Rnf2 in 15 hpf zebrafish embryos. No Rnf2 binding was detected on the promoters of these skeletal muscle genes (data not shown). The same result was shown in the 3 dpf Rnf2 ChIP-seq analysis reported by Chrispijn [31]. These data indicate that PRC1 represses the transcription of non-cardiac sarcomere genes in nascent hearts indirectly. PcG complexes attain transcription repression through histone modifications and chromatin compaction [45,46,47]. Different chromatin recruitment models of PRC1 and PRC2 have been shown in specific tissues and cells or biological processes. The classic model proposes that the recruitment of PRC1 depends on the prior presence of PRC2. PRC2 is first recruited to specific genomic loci and catalyzes H3K27me3. The H3K27me3 marks are recognized by the CBX component and then PRC1 is recruited via the chromodomain of CBX proteins [46,51,52]. Recently, an alternative recruitment model, proposing that PRC1 is targeted to chromatin without pre-existing PRC2 and H3K27me3, was proposed in X chromosome inactivation and mouse embryonic stem cells [20,25,53,54]. Conversely, the H2AK119ub deposited by PRC1 facilitates the subsequent recruitment of PRC2 [54]. The recruitment model of PRC complexes in the cardiac sarcomere assembly is still unclear. PRC2/EZH2 was reported to repress the transcription of skeletal muscle genes in developing heart by repressing the expression of Six1 [36,50]. We consulted the ChIP-seq data reported by Chrispijn and found low-level Rnf2 binding at the promoter region of *six1* [31], suggesting that Rnf2 may also restrain the expression of skeletal muscle genes in the heart by repressing *six1* transcripts. We also found high-level H3K27me3 enrichment at *six1* promoters, and the H3K27me3 enrichment was retained with Rnf2 deletion [31]. These indicated that both PRC1 and PRC2 complexes are targeted to the promoter region of *six1*, whereas the recruitment of PRC2 here is independent of PRC1. However, much more evidence is needed to confirm this speculation and to determine whether the recruitment of PRC1 depends on the pre-recruitment of PRC2, or whether each are targeted to chromatin independently.

3. PRC1 is involved in the construction of the zebrafish cardiac conduction system.

The main function of the CCS is to generate and conduct cardiac impulses to initiate and maintain rhythmic contractions. The impaired formation or malfunction of CCS components, including SAN dysfunction and AV blockage, can cause cardiac conduction disease (CCD), accompanied by severe arrhythmias [55]. The mammal CCS is mainly composed of pacemaker cardiomyocytes in SAN, AVN, and the ventricular conduction system (VCS) [10]. Although the zebrafish CCS features no AVN or VCS, it does feature slow-conducting AVC cardiomyocytes, which act as functional alternatives to mammalian AVN cardiomyocytes [10,56]. A better understanding of the development of the CCS’s components can help to illuminate the pathological and molecular mechanisms leading to CCD. Although knowledge related to the electrophysiological properties of CCS structures and functions has increased steadily since the anatomy of the CCS was identified 100 years ago, the molecular and genetic foundation of CCS specification and formation remain unclear [57]. Here, we demonstrated that Rnf2/PRC1 plays a critical role in the construction of the zebrafish CCS. The AVC formation was disrupted and the expression of AVC-specific markers, including *bmp4*, *vcana* and *alcama*, was expanded into flanked chambers instead of constricting in the AVC ring with loss of Rnf2 function. This indicates that PRC1 is required for the correct formation of CCS through the restriction of the AVC-specific transcriptional program. The CCS fulfills its function by propagating electrical impulses. The malformation of CCS components can induce changes of electrical signaling [58]. The decreased calcium signaling intensity in the hearts of Rnf2 mutants further confirms our conclusion. Our data provide more information on the genetic regulation of CCS construction and may facilitate the development of new treatment strategies for CCD.

Collectively, we generated a PRC1-null zebrafish line by knocking out the core PRC1 subunit, Rnf2. Our data indicate that PRC1 modulates cardiac sarcomere assembly by stabilizing sarcomere gene transcription programs, and regulates the specification of basic structures in the CCS by regulating the expression of AVC- and SAN-specific genes in the developing heart, which helps to maintain regular cardiac contraction. It is the first time that PRC1 has been reported to function in cardiac sarcomere assembly and cardiac conduction system construction during embryonic heart development. Our data offer a more detailed image of the function of PRC1 in vertebrate cardiac development. This may provide some new insights into the pathology of cardiac diseases caused by PRC1 loss-of-function.

## 4. Materials and Methods

### 4.1. Zebrafish Lines and Maintenance

AB line zebrafish were used in this study. The zebrafish were raised and maintained according to standard laboratory procedures as stated in the zebrafish book [59].

### 4.2. Generation of Rnf2 Mutants

The zebrafish Rnf2 mutants were generated using the CRISPR/Cas9 system. The guide RNA (gRNA) was designed by targeting the exon 3 of the *rnf2* gene. Embryos at the 1-cell stage were co-injected with 200 ng/µL *Cas9* mRNA and 80 ng/µL gRNA. The genomic DNA of 20 injected embryos at 24 hpf was extracted and subjected to PCR amplification. A DNA fragment containing the *rnf2* target site was amplified by PCR using the primers 5′-TTGAGGTAGTTGCTCCCAAAG-3′ and 5′-GGCATTCCTTGGTGGTCATA-3′, and the genotype was confirmed by DNA sequencing.

### 4.3. Western Blotting Experiments

The Western blot analysis was performed as previously described [60]. About 50 embryos were collected and dechorionated, and cell lysates were prepared using a TNE buffer (50 mM Tris-HCl (pH 8.0), 150 mM NaCl, 1% Triton X-100, 0.5% sodium deoxycholate, 5 mM EDTA, 1× DTT) with the cOmplete proteinase inhibitor (Roche, Switzerland). The lysates were resolved on 15% SDS polyacrylamide gels and immunoblotted with primary antibody anti-Rnf2 (A302-869A, Bethyl). The images were captured with an Image Quant LAS 4000mini (GE Healthcare Life Sciences, Chicago, IL, USA).

### 4.4. Whole Mount In Situ Hybridization (WISH)

The WISH was performed as previously described [61]. The DIG-labeled anti-sense probes were generated using a DIG RNA Labeling Kit (SP6/T7) (Roche, Switzerland). The DNA templates used to generate the probes were amplified by PCR and the primers used are listed in  Supplementary Table S1. For embryos at or after 36 hpf, the homozygotes were separated from their siblings according to their mobility and pectoral fin phenotype. For embryos before 36 hpf, as it was difficult to separate homozygous mutants from the heterozygous and wild-type siblings, WISH was performed for all self-cross progenies of *rnf2^+/−^* parents. After the WISH, each embryo was photographed and genotyped separately. The photographs were taken under a stereomicroscope (Leica Z16 APO) with a digital camera (Leica DFC450).

### 4.5. Histological Analysis

The zebrafish embryos at 4 dpf were fixed with Bouin’s solution at room temperature for 24 h. The fixed embryos were embedded in 1% agarose, dehydrated with gradient ethanol, hyalinized with xylene, and then embedded in paraffin. The paraffin-embedded embryos were sectioned, and the sections were stained with standard hematoxylin and eosin staining.

### 4.6. Quantitative Real-Time PCR

The relative mRNA levels of the genes in the *rnf2^−/−^* and control hearts were detected by using quantitative real-time PCR (qRT-PCR). The primers used are listed in Supplementary Table S2. Individual hearts from embryos were isolated for qRT-PCR analysis, as described below. To collect the individual hearts, the transgenic zebrafish strain *Tg* (*myl7: EGFP*) was used. The homozygous mutants were separated from their siblings according to their mobility and pectoral fin phenotype. The embryos were disrupted by pipetting up and down several times (depending on the developmental stages) with a 10 µL pipette. Next, the individual hearts were picked up by pipettes under a fluorescence stereomicroscope. The individual hearts were pooled into the same tube, and the total RNA was extracted using TransZol Up Plus RNA kit (Transgen, China). The cDNA was reverse-transcribed from the total RNA and used for qRT-PCR analysis.

### 4.7. Calcium Signaling Detection Using Fluo-4 AM

The calcium signal was detected according to the kit manual (Molecular Probes). Briefly, the hearts isolated from the 48 hpf *rnf2^−/−^* and wild-type embryos were transferred to a 20 mm glass-bottom cell culture dish containing external control solution (ECS, containing 140 mM NaCl, 4 mM KCl, 1.8 mM CaCl_2_, 1 mM MgCl_2_, 10 mM glucose, and 10 mM HEPES (pH 7.4)). Next, the embryos were incubated in an ECS medium containing 5 µM Fluo-4 AM, 10 µM blebbistatin, and 0.35 mM probenecid (Molecular Probes) for 30 min. The blebbistatin was added to uncouple the excitation-contraction process in the zebrafish embryonic hearts [62]. After being rinsed twice with ECS containing 0.35 mM probenecid, the hearts were transferred into ECS containing 0.35 mM probenecid. Images were taken with a confocal laser scanning microscope (Leica SP8 DLS).

### 4.8. Imaging, Quantification and Statistical Analysis

The area of pericardial edema of 72 hpf embryos was measured by marking the edema region on photos using ImageJ. The heart rates were counted manually under a dissecting microscope (WPI). The heart beating frequency in one minute per embryo was calculated manually. For the transmission electron microscopy of the cardiac muscle structure, the 80 hpf *rnf2^−/−^* and wild-type embryos were fixed, embedded in the Spur resin, and then sectioned. The sections were stained with uranyl acetate and lead citrate. The images were taken with a Hitachi TEM system (Japan). The data were analyzed with the GraphPad Prism 7.0 software. The values are presented as mean ± SEM. The *p*-values were calculated using two-tailed Student’s test, * *p* ≤ 0.05, ** *p* ≤ 0.01, *** *p* ≤ 0.001.

## Supplementary Materials

The following are available online at https://www.mdpi.com/article/10.3390/ijms222111368/s1.
