# Supplementary material for: PRC1 Stabilizes Cardiac Contraction by Regulating Cardiac Sarcomere Assembly and Cardiac Conduction System Construction
Source: Int J Mol Sci. 2021 Oct 21;22(21):11368. doi: 10.3390/ijms222111368 (PMC8583368; doi:10.3390/ijms222111368)
Supplement: Supplementary file 1 [file ijms-22-11368-s001.zip › ijms-1404443-supplementary.pdf]

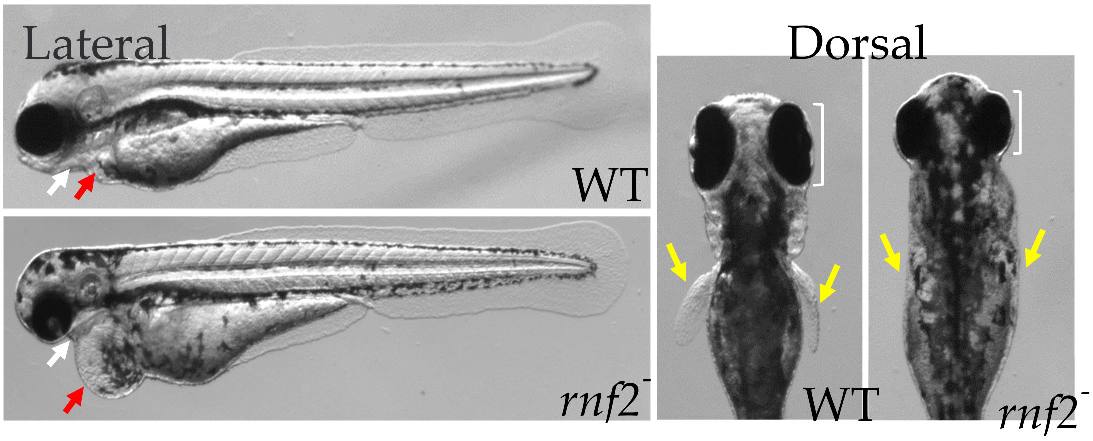

Figure S1. Phenotypes of rnf2-/- and wild type embryos at 3 dpf. The defects include cranial facial defects (white arrows), pericardial edema (red arrows, absent of pectoral fin (yellow arrows) and small eyes (white brackets).
